# Insight into the broadened substrate scope of nitrile hydratase by static and dynamic structure analysis†

**DOI:** 10.1039/d2sc02319a

**Published:** 2022-07-06

**Authors:** Dong Ma, Zhongyi Cheng, Lukasz Peplowski, Laichuang Han, Yuanyuan Xia, Xiaodong Hou, Junling Guo, Dejing Yin, Yijian Rao, Zhemin Zhou

**Affiliations:** Key Laboratory of Industrial Biotechnology (Ministry of Education), School of Biotechnology, Jiangnan University Wuxi Jiangsu 214122 China raoyijian@jiangnan.edu.cn zhmzhou@jiangnan.edu.cn; Institute of Physics, Faculty of Physics, Astronomy and Informatics, Nicolaus Copernicus University in Torun Grudziadzka 5 87-100 Torun Poland; Jiangnan University (Rugao) Food Biotechnology Research Institute Rugao Jiangsu China

## Abstract

The narrow substrate scope limits the wide industrial application of enzymes. Here, we successfully broadened the substrate scope of a nitrile hydratase (NHase) through mutation of two tunnel entrance residues based on rational tunnel calculation. Two variants, with increased specific activity, especially toward bulky substrates, were obtained. Crystal structure analysis revealed that the mutations led to the expansion of the tunnel entrance, which might be conducive to substrate entry. More importantly, molecular dynamics simulations illustrated that the mutations introduced anti-correlated movements to the regions around the substrate tunnel and the active site, which would promote substrate access during the dynamic process of catalysis. Additionally, mutations on the corresponding tunnel entrance residues on other NHases also enhanced their activity toward bulky substrates. These results not only revealed that residues located at the enzyme surface were a key factor in enzyme catalytic performance, but also provided dynamic evidence for insight into enzyme substrate scope broadening.

## Abstract 2

Mutations of two gating residues at the substrate access tunnel entrance direct the substrate scope of NHases.

## Graphical abstract

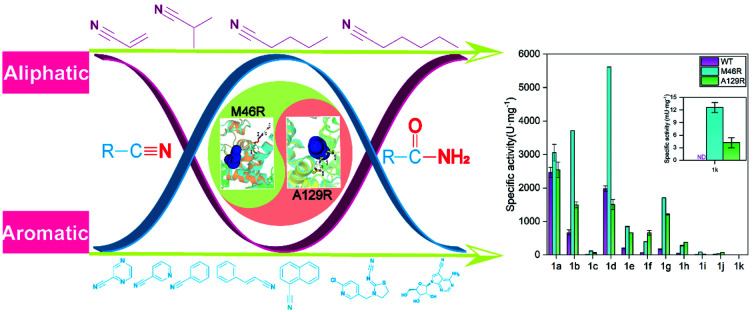

## Introduction

For centuries, traditional chemical catalysis has dominated the synthesis of valuable compounds. However, with biocatalysis being a promising and environmentally friendly alternative over the past few decades, traditional chemical catalysis is becoming overwhelmed by its green counterpart.^1^ Although biocatalysis has unparalleled advantages, its applications in some fields are hindered by the narrow substrate scope and low catalytic activity. To extend the application of certain enzymes, it is particularly important to broaden the substrate scope. Efforts have been made to identify enzymes with a broad substrate scope using screening methods such as gene mining,^2,3^ but the obtained enzymes cannot always meet the laboratory or industry requirements. A second option for expanding the substrate scope is to modify the existing enzymes.^4^

Traditional substrate scope engineering of a particular enzyme involves modifying the substrate-binding pocket and substrate access tunnel.^5^ For example, through elucidation of the gating characteristics and constitution of the substrate access tunnel, it was determined that threonine deaminase increased catalytic efficiency towards bulky substrates 90.6-fold.^6^ Mutations on the substrate access tunnel forming residues, V333 and T334, of prodigiosin ligase PigC, which involve the translocation of small pyrroles, enhanced the catalytic efficiency more than 45-fold.^7^ On mutating two bottleneck residues in the cytochrome P450_Bsβ_HI substrate access tunnel, its decarboxylation activity increased by 15.2-fold.^8^ Therefore, applying the existing knowledge and strategy to modify the substrate-binding pocket and access tunnels is promising for refining the substrate scope of enzymes.

Nitrile hydratase (NHase, EC 4.2.1.84), comprising α- and β-subunits, is a metalloenzyme that catalyzes nitriles into amides.^9^ It has been widely applied in the industrial production of acrylamide and nicotinamide and shows great potential in the future production of high-value amide products.^10^ However, the activities of NHases towards bulky nitriles such as thiacloprid and toyocamycin are very low.^11,12^ Therefore, further industrial applications for producing various valuable amides using NHase are strictly hindered by their narrow substrate scope, and academia and industry require impressive tailor-made NHases. The NHase active site contains either a non-heme iron or non-corrin cobalt,^13^ buried in a deep internal pocket,^14^ similar to most enzymes in nature. Hence nitrile substrates should pass through a tunnel to access the active site, and rational tunnel engineering might be a plausible choice for NHase substrate scope modification. Modifying the bottleneck region of the substrate access tunnel of NHases has been reported to result in the well-tailored regio- and stereo-selectivity of NHases.^15,16^ Therefore, the catalytic performance of NHase toward bulky nitrile substrates can also be improved through the tunnel engineering strategy. However, the current tunnel engineering strategies mainly focus on the internal regions inside the tunnel space, and the influence of the entrance region at the substrate access tunnel surface on the catalytic performance has not yet drawn enough attention.

In this study, we discovered that two gating residues at the substrate access tunnel entrance play a key role in expanding the substrate scope of NHases, especially for bulky nitrile substrates. We rationally refined the tunnel entrance region of NHase from *Pseudonocardia thermophila* JCM 3095 (*Pt*NHase), based on substrate access tunnel calculations. Two successful mutants, *Pt*NHase-βM46R and *Pt*NHase-βA129R, which exhibited high catalytic activity toward bulky substrates were obtained. Structural analysis revealed that the mutation of the gating residues introduced anti-correlated movements to the substrate access tunnel, resulting in a “breathing mode” of the tunnel entrance. These results not only revealed that residues located at the enzyme surface were a key factor in enzyme catalytic performance, but also provided dynamic evidence for insight into enzyme substrate scope broadening.

## Results and discussion

### Low catalytic performance of *Pt*NHases toward bulky nitrile substrates

**Fig. 1:**
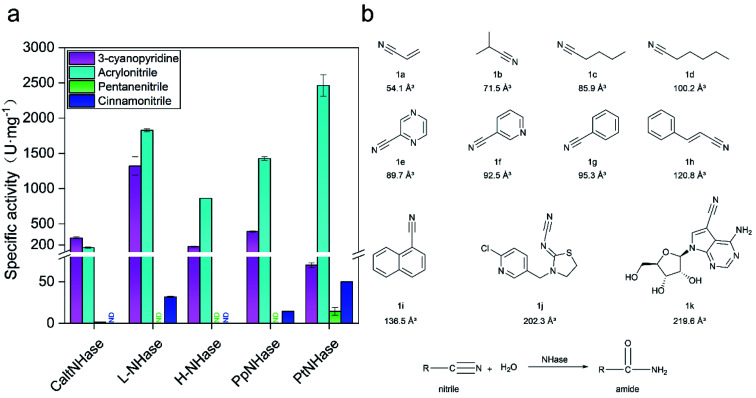
Low activity of NHases toward bulky nitriles. (a) Activity of NHases toward two regular nitriles (3-cyanopyridine and acrylonitrile) and two relatively bulky nitriles (pentanenitrile and cinnaonitrile). *Calt*NHase represents NHase from *Caldalkalibacillus thermarum*; L-NHase and H-NHase represent low and high molecular weight nitrile hydratase from *Rhodococcus rhodochrous* J1, respectively; *Pp*NHase stands for NHase from *Pseudomonas putida*; *Pt*NHase represent NHase from *Pseudonocardia thermophila.* ND: not detected. (b) Substrates used in the present study. The volumes of the substrates were calculated using VolumeCalculator. Herein, 1a (acrylonitrile), 1b (isobutyronitrile), 1c (pentanenitrile), 1d (hexanenitrile), 1e (2-cyanopyrazine), 1f (3-cyanopyridine), 1g (benzonitrile), 1h (cinnamonitrile), 1i (1-naphthonitrile), 1j (thiacloprid) and 1k (toyocamycin) were selected as substrates. All experiments were performed in triplicate, and error bars indicate ± sd.

In general, NHases exhibit high activity toward regular nitrile substrates such as 3-cyanopyridine and acrylonitrile whereas their activity toward bulky substrates is very low.^13^ In the present study, five NHases from different strains that were applied or have the potential to be applied in industrial amide production were tested using two regular nitrile substrates (acrylonitrile 1a and 3-cyanopyridine 1f) and two relatively bulky nitrile substrates (pentanenitrile 1c and cinnamonitrile 1h). All the tested NHases exhibited high or moderate activity toward 3-cyanopyridine and acrylonitrile (Fig. 1a); however, their activities toward the bulky substrates pentanenitrile and cinnamonitrile were extremely low. Given the best catalytic performance among the tested NHases, and its intrinsic thermophilic characteristic,^17,18^ the NHase from *Pseudonocardia thermophila* JCM3095 (*Pt*NHase) was chosen for further study.

In order to broaden the substrate scope of *Pt*NHase, structural information is necessary. The crystal structure of *Pt*NHase (PDB ID: 1IRE) is available in the Protein Data Bank (PDB), and its substrate access tunnel has been identified.^19,20^ For a bulky substrate to be catalyzed by an enzyme, the substrate must firstly migrate through the access tunnel and then be accommodated inside the catalytic cavity. Static PDB structure analysis using the CAVER tool showed that the volume of the WT active site cavity was 159.1 Å^3^, which was larger than that of bulky substrates such as cinnamonitrile (1h, 120.8 Å^3^) and 1-naphthonitrile (1i, 136.5 Å^3^) (Fig. 1b). Therefore, the low activity of *Pt*NHase toward the two bulky nitrile substrates might be caused by migration being hindered through the narrow access tunnel, particularly through the entrance region at the tunnel surface.

### Tunnel entrance engineering for broadening the substrate scope of *Pt*NHase

**Fig. 2:**
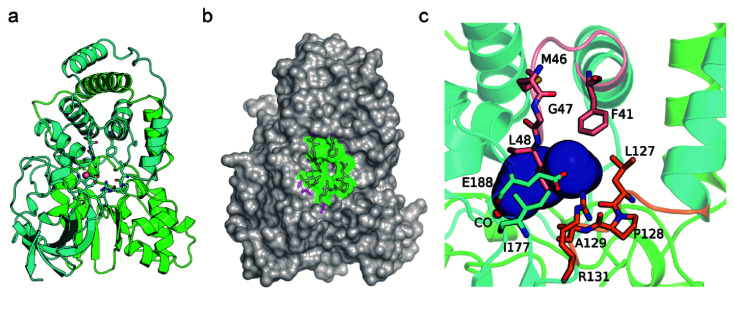
Identification of the residues at the entrance of the substrate access tunnel of *Pt*NHase. (a) Crystal structure of the WT *Pt*NHase dimer. (b) The entrance region of the substrate tunnel. (c) The inside of the entrance region of the substrate tunnel. The entrance region comprises 10 amino acid residues, βPhe41, βMet46, βGly47, βLeu48, βLeu127, βPro128, βAla129, βArg131, βIle177 and βGlu188, existing in an “upper entrance loop” (salmon) and a “lower entrance loop” (orange). The substrate tunnel are indicated by blue surface.

The CAVER software was used to analyze the substrate access tunnel of *Pt*NHase. The length of the calculated tunnel was 12.8 Å, and the entrance region at the enzyme surface comprised ten amino acid residues which were mainly distributed on an “upper entrance loop” and a “lower entrance loop”: βPhe41, βMet46, βGly47, βLeu48, βLeu127, βPro128, βAla129, βArg131, βIle177, and βGlu188 (Fig. 2 and S2†).

Studies have reported that conserved residues might play important roles in directing the activity of a certain enzyme.^21–23^ To exclude conserved residues from the entrance region and choose the optimal size of the randomization site and minimize the labor-determining screening effort, conservation analysis was performed using the ConSurf Server.^24^ Five amino acid residues (βPhe41, βMet46, βLeu127, βPro128, and βAla129) with conservation grades lower than seven were chosen as hotspots for mutant library construction (Fig. S3†). Seven amino acid residues, including polar and nonpolar residues with side chains of different sizes (alanine, leucine, phenylalanine, glutamic acid, histidine, lysine and glutamine), were introduced to construct mutants at the selected sites.

**Fig. 3:**
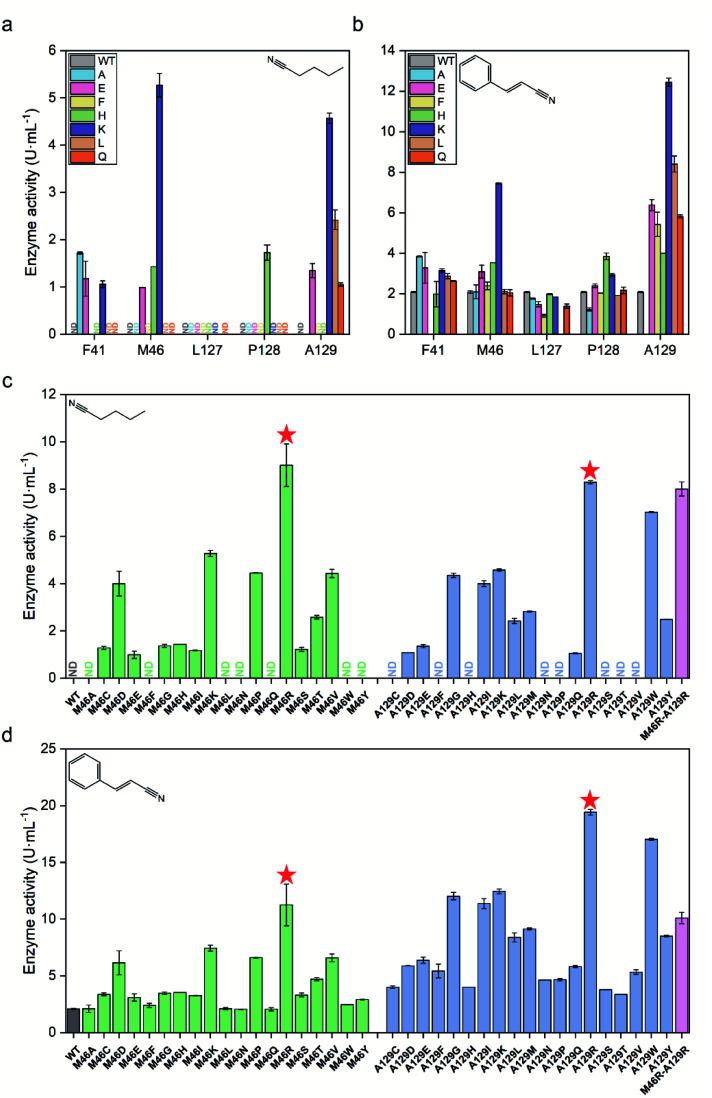
Screening of mutant NHases exhibiting high activity toward pentanenitrile and cinnamonitrile. (a) Preliminary activity screening of potential hotspots toward pentanenitrile. (b) Preliminary activity screening of potential hotspots toward cinnamonitrile. (c) Activity of the saturated mutants at positions βM46 and βA129 toward pentanenitrile. (d) Activity of the saturated mutants at positions βM46 and βA129 toward cinnamonitrile. ND, not detected. All experiments were performed in triplicate, and error bars indicate ± sd.

The activity of each mutant was evaluated after incubation with pentanenitrile and cinnamonitrile as substrates. As shown in Fig. 3a and b, mutants of *Pt*NHase-βM46K and *Pt*NHase-βA129K exhibited significantly increased activity toward both substrates, indicating that residues βMet46 and βAla129 at the tunnel entrance region play the most crucial roles in directing the catalytic performance. Subsequently, site-directed saturation mutagenesis was performed at these two sites. Based on the activity of the constructed mutants toward both substrates, two mutants, *Pt*NHase-βM46R and *Pt*NHase-βA129R, which exhibited the highest activity, were obtained (Fig. 3c and d). Although the combinational double-point mutant *Pt*NHase-βM46R/βA129R was constructed, it exhibited lower activity than that of the two single mutants (Fig. 3c and d). Therefore, further analysis mainly focused on *Pt*NHase-βM46R and *Pt*NHase-βA129R.

### Characterization of *Pt*NHase-βM46R and *Pt*NHase-βA129R variants

The two mutants were then purified (Fig. S4†) and characterized using pentanenitrile and cinnamonitrile as substrates. As shown in Table 1, introducing β46R and β129R mutations into *Pt*NHase resulted in an 8.2-fold and 4.2-fold increase in activity toward pentanenitrile, and a 6.2-fold and 8.3-fold increase in activity toward cinnamonitrile, respectively. To better investigate the substrate scope of the two mutants, their specific activities toward the other nine nitrile substrates were measured. As shown in Table 1, the activity of the WT toward different substrates decreased significantly as the substrate size increased, whereas the catalytic performance of *Pt*NHase-βM46R and *Pt*NHase-βA129R was significantly improved (from 1.6- to 14.6-fold). Interestingly, when bulky sangivamycin was used as a substrate, the WT enzyme activity was not detected, whereas both mutants exhibited detectable catalytic activity. These results indicated that the substrate scope of *Pt*NHase was successfully broadened.

**Table 1:** Specific activity of *Pt*NHase toward different nitrile substrates

Substrate	Specific activity (U mg^−1^)				
	WT	M46R	Fold	A129R	Fold
Acrylonitrile	2463 ± 153.8	3056 ± 251.3	1.2	2542 ± 236.8	1
Isobutyronitrile	666.2 ± 80.8	3720.5 ± 77.3	5.6	1498.9 ± 89.6	2.3
Pentanenitrile	14.4 ± 0.9	118.4 ± 3.7	8.2	60.5 ± 15.3	4.2
Hexanenitrile	1982.4 ± 83.8	5613.3 ± 112.9	2.8	1503.6 ± 154.8	0.8
2-Cyanopyrazine	203.2 ± 8.1	851.2 ± 8.1	4.2	660.4 ± 4.0	3.3
3-Cyanopyridine	66.7 ± 1.0	396.4 ± 8.3	5.9	661.8 ± 69.0	9.9
Benzonitrile	176.5 ± 8.1	1704.7 ± 11.2	9.7	1211.9 ± 17.6	6.9
Cinnamonitrile	45.8 ± 1.0	282.9 ± 12.3	6.2	379.1 ± 5.2	8.3
1-Naphthonitrile	13.1 ± 1.7	84.3 ± 4.2	6.4	20.6 ± 1.8	1.6
Thiacloprid	5.4 ± 0.3	42.2 ± 1.0	7.8	78.9 ± 2.3	14.6
Toyocamycin	0	12.6 ± 1.2^a^	—	4.2 ± 1.2^a^	—

a mU mg^−1^. Herein, “mU” refers to the amount of enzyme that 1 μmol of substrate consumed per hour at 25 °C.

The kinetic parameters of mutants βM46R and βA129R toward substrates 1c, 1f and 1h were determined (Table S4 and Fig. S5†). With respect to the biocatalysis of substrate 1f, the *K*_m_ values of βM46R and βA129R were 2.2- and 1.7-fold lower, respectively, than that of their parent enzyme. The *k*_cat_ values of the corresponding mutants increased by 3.1- and 3.8-fold, respectively. Accordingly, the *k*_cat_/*K*_m_ values of these two variants increased by 6.8- and 6.3-fold, respectively. When using 1c or 1h as a substrate, both mutants βM46R and βA129R had slightly lower *K*_m_ values compared to that of the wild-type, and their *k*_cat_ values improved to varying degrees, among which βM46R showed 2.8- and 3.6-fold higher *k*_cat_ values for 1c and 1h while βA129R showed 1.2-fold and 3.5-fold higher *k*_cat_ values for 1c and 1h, respectively.

It has been reported that a stability–activity trade-off might occur during the evolutionary journey of a certain enzyme.^25^*Pt*NHase is a thermophilic enzyme; however, its stability might decrease with improved activity toward various substrates after mutagenesis. Therefore, the thermal stability of the two mutants was compared with that of the WT. The three enzymes were treated at 50 °C for 3 h, and their residual activities were tested using 3-cyanopyridine as a substrate (Fig. S6†). The residual activities of the WT and *Pt*NHase-βM46R were 64.3% and 62.0%, respectively, indicating that the mutation at the βMet46 site did not significantly affect its thermostability. However, the *Pt*NHase-βA129R mutant lost more than half of its initial activity, suggesting that it is a stability–activity trade-off mutant. Based on these results, in addition to a delicate balance of stability and flexibility, there are both theoretical and empirical reasons to believe that the relationship between these traits may be more complicated.

### Insights into the broadened substrate scope by static structure analysis

**Fig. 4:**
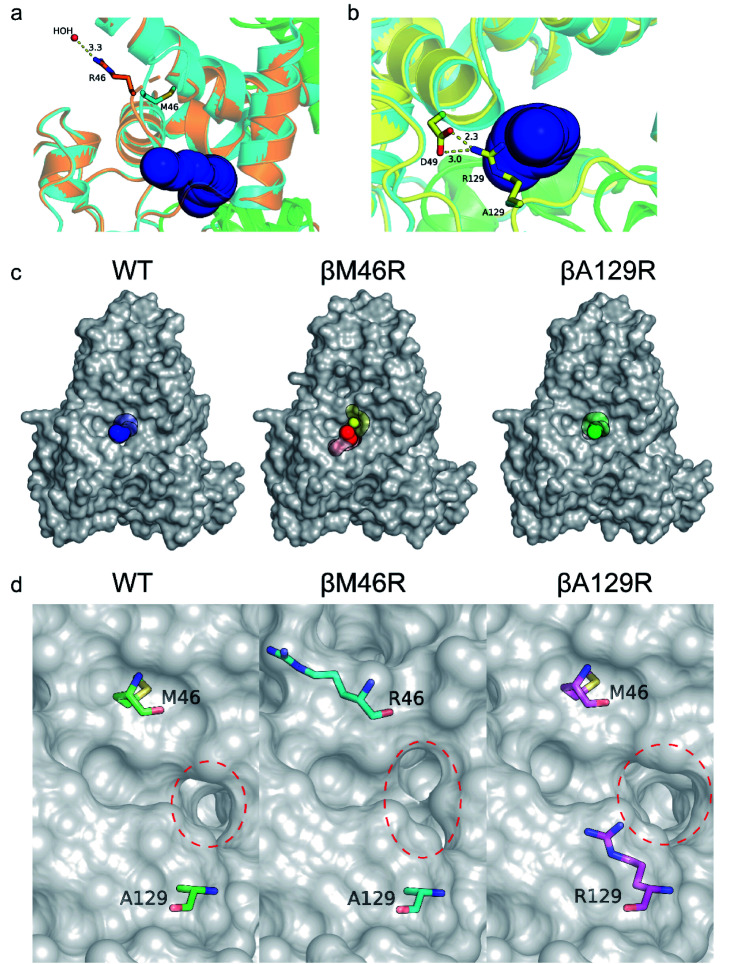
Crystal structure analysis of WT, *Pt*NHase-βM46R and *Pt*NHase-βA129R. Entrance comparison of WT with (a) *Pt*NHase-βM46R and (b) *Pt*NHase-βA129R. (c) Surface of substrate access tunnels of WT and the two mutants. (d) The entrance of substrate access tunnels of WT and the two mutants. The entrances are highlighted in red dashed circles. The relevant residues are presented as colored sticks.

Several studies have shown that tunnel engineering with small amino acid substituents can improve the catalytic performance of enzymes as the introduction of amino acid residues with small side chains can expand the tunnel space.^6,26^ In the present study, however, it is quite interesting that two bulky amino acid residues were introduced into the entrance region of the substrate access tunnel, which significantly increased the activity of *Pt*NHase toward several bulky substrates. To understand the mechanism underlying the expanded substrate scope with bulky amino acid substituents, the crystal structures of *Pt*NHase-βM46R and *Pt*NHase-βA129R were obtained and analyzed. Both structures had clear electron density at the mutated sites. The βArg46 and βArg129 residues were located at the “upper entrance loop” and “lower entrance loop” of the substrate access tunnel, respectively. Based on the structural alignment of WT and *Pt*NHase-βM46R (PDB ID: 7W8L), the side-chain orientations of the β46 site differed tremendously. The hydrophobic side chain of βMet46 in WT was buried and oriented towards the interior of the protein, whereas the bulky side chain of βArg46 in *Pt*NHase-βM46R formed a hydrogen bond with a water molecule, showing an obvious outward offset to the solvent (Fig. 4a). In the case of *Pt*NHase-βA129R, the side chain of βArg129 formed a salt-bridge with βAsp49 (Fig. 4b), which maintained the side chain of the Arg129 outward offset from the substrate access tunnel. No interactions were observed between β129A and its surrounding residues in the WT, and the side chain of β129A was also buried and oriented towards the interior of the protein. These results indicate that the outward offset of the mutants may maintain a more conducive migration of the bulky substrates, while the orientation towards the interior of the protein of the side chains would cause a stereospecific blockade, hindering the entry of bulky substrates. This hypothesis is supported by the fact that most of the hydrophobic amino acid-substituted mutants did not show improved activity toward pentanenitrile and cinnamonitrile (Fig. 3c and d).

To further investigate the influence of the mutated sites on the substrate access tunnel entrance, tunnel calculations were performed using the CAVER software. Compared with the WT, an additional potential tunnel that started from the active site and ended at the surface entrance of the *Pt*NHase-βM46R mutant was observed (Fig. 4c), which might provide more alternatives for bulky substrate migration. In addition, entrance shape and size analysis indicated that the entrance of the *Pt*NHase-βA129R was expanded compared to that of its parent enzyme (Fig. 4d). The changes in the entrance of the substrate tunnel might be conducive to bulky substrate entry, resulting in substrate scope broadening.

### Insights into the broadened substrate scope by dynamic structure analysis

**Fig. 5:**
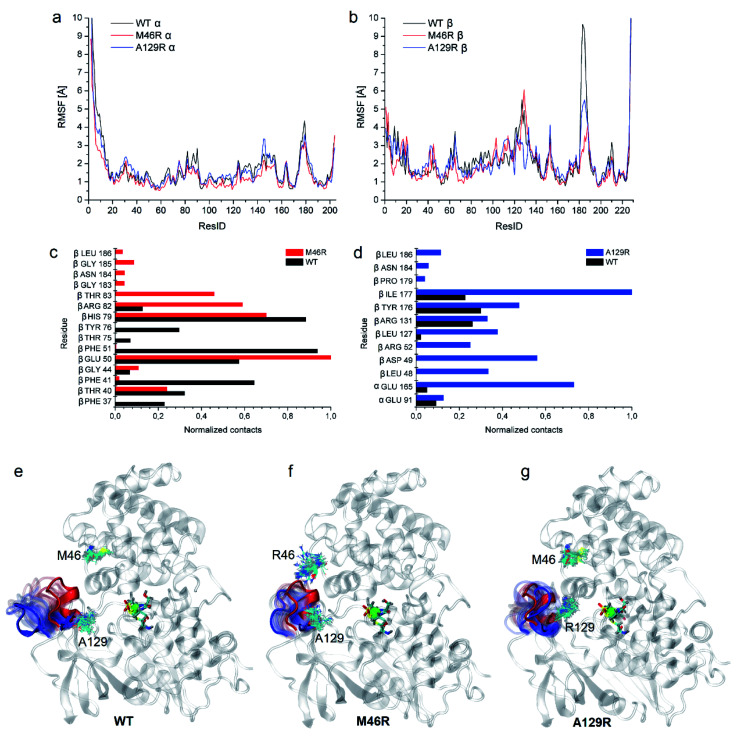
Dynamic analysis of WT and the two mutants. (a) RMSF comparison of the α subunit of WT and the two mutants. (b) RMSF comparison of the β subunit of the WT enzyme and the two mutants. (c) Close contacts of residue β46 with its neighbor residues in WT and *Pt*NHase-βM46R. (d) Close contacts of residue β129 with its neighbor residues in WT and *Pt*NHase-βA129R. Process of the 180–184 region in case of (e) WT, (f) *Pt*NHase-βM46R, and (g) *Pt*NHase-βA129R. The red α-helix corresponds to the starting structure, blue to the structure after 200 ns. Transparent positions were recorded every 10 ns. The mutated amino acids during simulations are shown (snapshots every 10 ns).

To clarify the dynamic effects and validate the structural rationalization, 200 ns molecular dynamics (MD) simulations of the WT and the two variants were performed based on the crystal structures. Root mean square deviations (RMSD) from 200 ns simulations were calculated for each dimer of the NHase tetramer (Fig. S7†). The RMSD values of both dimers were about 3 Å or lower which is typical of globular proteins. Analysis of root mean square fluctuations (RMSF) showed that fluctuations of the α subunit were very similar, and obvious differences were observed in the region of β181 to β187, where the RMSF values of the two mutants were much lower than those of the WT (Fig. 5a and b). The dynamic stabilization process of this region in the two mutants was thus calculated and compared to those of the WT; the close contacts (within 4 Å) of β46 and β129 with their neighboring residues are shown in Fig. 5c and d. Additional contacts of β46 with the residues βGly183, βAsn184, βGly185, and βLeu186, and β129 with the residues βPro179, βAsn184, and βLeu186 in this region were observed in the two variants, whereas no contact was observed in WT (Fig. 5c and d). These results revealed that the interactions caused by the two mutant residues stabilized the β181 to β187 region. This conclusion was also supported by the results of the structure dynamic process, as shown in Fig. 5e, f and g, and a marked change in the β181 to β187 region (α-helix) of the WT was observed after 200 ns, while this region in the two variants kept relatively stable; either β46R in *Pt*NHase-βM46R or β129R in *Pt*NHase-βA129R located close to this region rather than those amino acid residues in the WT.

**Fig. 6:**
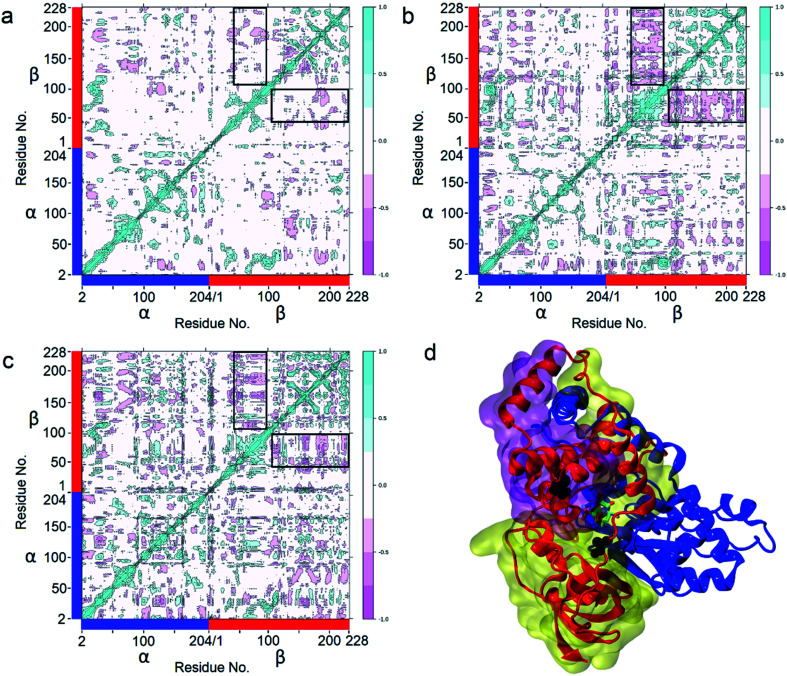
Determination of Dynamic Cross-Correlation Matrices (DCCMs) based on 200 ns simulations. (a) WT; (b) *Pt*NHase-βM46R; (c) *Pt*NHase-βA129R. The two parts with the highest anticorrelated movement (residues β37–97 and residues β107–227) are highlighted in black panes. (d) The two parts with the highest anticorrelated movement in structures. The transparent purple surface represents residues β37–97, and the transparent yellow surface represents residues β107–227. Met46 and Ala129 are shown as black spheres. The α subunit is represented by a blue ribbon, and the β subunit is represented by a red ribbon.

Enzyme catalysis is a dynamic process, during which the enzyme structure usually changes to a certain extent. To clarify the effect of the mutation on the dynamic process, dynamic cross-correlation matrix (DCCM) information analyses were performed to check the amino acid residue movement correlations based on MD simulations.^27,28^ Compared with the weak correlations and anti-correlation observed in the WT (Fig. 6a), strong anti-correlated movements were observed between residues 37–97 and 107–227 in the β subunit of the two mutants (Fig. 6b and c). The strong anti-correlated movements in the β subunit of the two mutants might maintain the trade-offs of the inflexibility of the mutant enzymes because the stability of a protein needs flexibility for free energy release. These anti-correlated movements indicate that these two parts of the protein generally move apart or move towards each other, similar to breathing. The two mutated sites of *Pt*NHase-βM46R and *Pt*NHase-βA129R located in the region of β37–β97 and β107–β227, respectively, and the substrate tunnel and the active site of the mutants would open widely during the breathing mode, resulting in an expanded substrate access tunnel together with broadened active site cavities (Fig. 6d). The active-site cavities and substrate access tunnel entrance were calculated using Caver.^29^ Ten structures were selected every 20 ns from the 200 ns simulations for each variant. The cavity volume for WT was 178.2 ± 55.0 Å^3^, while in *Pt*NHase-βM46R and *Pt*NHase-βA129R, they were 193.7 ± 40.4 Å^3^ and 206.9 ± 61.3 Å^3^, respectively. The maximum entrance sizes of WT, *Pt*NHase-βM46R, and *Pt*NHase-βA129R were estimated to be 39 Å^2^, 47 Å^2^, and 46 Å^2^, respectively. The broadened active site cavities and substrate access tunnel entrance in the two mutants might allow bulky nitrile substrates to approach the active site for catalysis easily.

### Stability analysis *via* Gibbs free energy (ΔΔG) calculation

Proteins are highly dynamic, metastable molecular machines. A protein conformation with low free energy exists in a dynamic process, and the conformation would be changed with the effect of the surrounding environment, during which the flexible regions could release free energy to keep the protein in a stable conformation. Therefore, the flexible regions of a protein are important for protein stability. Usually the flexibility reduction in one flexible region due to protein mutation leads to a new flexible region.^30^ However, it is inscrutable that the RMSF values of the β181 to β187 region in the two mutants were much lower than those of the WT, while the RMSF values in other regions were very similar. In addition, stability and activity are generally found to be in a trade-off relationship,^25^ where activity improvement usually leads to stability reduction. Gibbs free energy (ΔΔG) calculation based on DynaMut2, and assessing changes in stability and flexibility upon single and multiple point missense mutations,^31^ indicated that compared with the WT, the ΔΔG of the two variants were −0.95 and −0.62 kcal mol^−1^, respectively, a stability reducing result (Fig. S8†). However, though the mutant *Pt*NHase-βA129R exhibited low thermostability, the mutation *Pt*NHase-βM46R kept its thermostability similar to WT. These findings indicated that it is possible that there are other factors contributing to the release of free energy instead of the flexible regions.

### The general significance of gating residues on the catalytic performance

As the amino acid residues at sites β46 and β129 play key roles in the catalytic performance of *Pt*NHase toward various nitrile substrates, it is intriguing to know whether the same positions of other NHases have similar functions in their catalytic efficiency. It is known that H-NHase from *Rhodococcus rhodochrous* J1 has been used for the industrial production of acrylamide and nicotinamide, and also their formation mechanism was well studied.^32,33^ Besides, thermophilic NHase from the extremophile *Caldalkalibacillus thermarum* TA2.A1 (*Calt*NHase) also has great potential in amide production because of its excellent stability.^34,35^ Tailoring the amino acid forming the substrate access tunnel entrance of these NHases might further improve their catalytic performance. Therefore, based on amino acid sequence alignment (Fig. S9†), site-directed mutagenesis was performed at the corresponding sites of H-NHase and *Calt*NHase. The corresponding amino acid residues of β46 and β129 were confirmed to be S46 and L133 in H-NHase, and Y46 and T128 in *Calt*NHase, respectively, based on the structure analysis.

The specific activities of the purified mutants toward cinnamonitrile were determined (Fig. S11†). Three mutant H-NHases, corresponding to *Pt*NHase-βM46R, showed higher catalytic activity, varying from 1.6- to 2.5-fold compared to that of their parent enzyme, and four mutant H-NHases, corresponding to *Pt*NHase-βA129R, exhibited increased specific activity varying from 1.4- to 2.2-fold. The activities of the mutants of *Calt*NHase corresponding to *Pt*NHase-βM46R and *Pt*NHase-βA129R increased from 1.4- to 6.8-fold, respectively. These results demonstrate that the two gating sites located at the entrance of the substrate access tunnel in other NHases also play important roles in directing activity toward bulky nitrile substrates.

## Conclusions

Here we reported that the mutation of two amino acid residues at the NHase surface led to significant substrate scope broadening. The mutant enzymes exhibited an obviously expanded substrate scope, especially toward bulky nitrile substrates. This phenomenon was also observed in another enzyme, d-carbamoylase, in which a loop located at the surface of the substrate entrance tunnel also plays an important role in its catalytic performance.^26^ Based on the expanded substrate scope resulting from the mutation of the two gating residues in NHases and the same phenomenon observed in d-carbamoylase, we strongly demonstrated that the amino acid residue located at the entrance of the substrate access tunnel is a key factor in enzyme catalytic performance. Moreover, we clarified that a dynamic structure analysis is more exact for insight into enzyme catalytic performance. These findings above provide valuable information for academic research and industrial applications of other enzymes.

## Experimental section

### Chemicals and reagents

Substrates 1a (acrylonitrile), 1b (isobutyronitrile), 1c (pentanenitrile), 1d (hexanenitrile), 1e (2-cyanopyrazine), 1f (3-cyanopyridine), 1g (benzonitrile), 1h (cinnamonitrile), 1i (1-naphthonitrile), 1j (thiacloprid), 1k (toyocamycin) and the corresponding amides were purchased from Macklin Co., Ltd (Shanghai, China). Tryptone and yeast extract were purchased from Thermo Fisher Scientific (Waltham, MA, USA). NaCl, KCl, Na_2_HPO_4_·12H_2_O, K_2_HPO_4_, KH_2_PO_4_, NaOH, and CoCl_2_·6H_2_O were obtained from Sinopharm Chemical Reagent Co., Ltd (Shanghai, China). Kanamycin, dithiothreitol, and isopropyl β-d-thiogalactoside (IPTG) were purchased from Sangon Biotech (Shanghai, China). Desthiobiotin was purchased from Sigma-Aldrich (St. Louis, MO, USA). Other commercial reagents like standards and solvents were purchased from Aladdin Reagents (Shanghai, China), Macklin (Shanghai, China), Takara (Dalian, China), and Sinopharm Chemical Reagent Co., Ltd, respectively.

### Construction and screening of the mutation library

An expression plasmid containing alpha, beta and regulatory protein genes with pET-24a as the vector was constructed by our group (Fig. S1†). The beta, alpha and regulatory protein genes in sequence share a single T7 promoter and separate ribosome binding sites, and the Strep-tag II is located at the C-terminus of the beta subunit. Mutations in *Pt*NHase were created by site-directed mutagenesis using a whole-plasmid approach.^36,37^ The primers are listed in ESI Table 2.† Mutations were verified by DNA sequencing and transformed into *E. coli* BL21(DE3) cells. Single colonies were picked and grown in 5 mL LB medium (10 g tryptone, 5 g yeast extract, 10 g NaCl) containing 50 μg mL^−1^ kanamycin at 37 °C. Next, CoCl_2_·6H_2_O and IPTG were added to a final concentration of 0.1 g L^−1^ and 0.4 mM when the OD_600_ value reached 0.6. After induction at 24 °C for 16 h, the cells were harvested by centrifugation at 10 000*g* for 5 min, and the pellets were washed and resuspended 2–3 times with 10 mM kalium phosphate buffer (KPB) (containing 0.01 M K_2_HPO_4_ and 0.01 M KH_2_PO_4_, 4 : 1 mixed, pH 7.4). To screen efficient variants initially, enzyme activity assays were firstly performed using a certain number of whole cells for 1c and 1h at 25 °C for 10 and 5 min in a metal bath thermostat, respectively (Table S3†).

### Enzyme purification

After incubation in an ice bath, the suspensions of the selected variants were sonicated using an ultrasonic cell disruptor for 30 min (750 W, 42% Ampl, 3 s on 7 s off). Subsequently, the supernatant was collected by centrifugation at 12 000*g* for 30 min. The supernatant samples were filtered with a 0.22 μm water-based filter and loaded onto a 1 mL StrepTrap column (GE Healthcare UK Ltd), and the enzyme was isolated with an AKTA purifier using an isocratic elution of 2.5 mM desthiobiotin in PBS buffer (20 mM Na_2_HPO_4_·12H_2_O, 280 mM NaCl, 6 mM KCl). The target enzyme samples were pooled, and the concentration was adjusted to 0.5 mg mL^−1^ with the Bradford assay. Purity was checked using sodium dodecyl sulfate–polyacrylamide gel electrophoresis. Purified enzymes were stored in aliquots at −80 °C in PBS buffer at pH 7.4.

### Enzyme activity and kinetic parameter determination

*Pt*NHase activity was assayed using procedures performed previously.^38^ Enzyme activity was assayed by measuring the amount of amide product generated from the nitrile substrate. The reaction mixture of a total volume of 0.5 mL contained 10 mM KPB (pH 7.4), a certain substrate concentration, and 10 μL of appropriately purified enzyme. The reaction was performed at 25 °C for an appropriate time and terminated by adding acetonitrile (0.5 mL). The amount of product formed in the reaction mixture was determined using an HPLC (Hitachi) equipped with a C18 reverse-phase column. Acetonitrile : water (1 : 2) was used as the mobile phase. The liquid phase detection conditions, including the flow rate, collection wavelength, and time, are listed in ESI Table 3.† One unit (U) of enzyme activity was defined as the amount of enzyme required to release 1 μmol of product by catalyzing the substrate per minute at 25 °C. Additionally, one milliunit (mU) of enzyme activity for the catalysis of toyocamycin is defined as the amount of enzyme that 1 μmol of substrate consumed per hour at 25 °C.

The kinetic parameters (*K*_m_, *k*_cat_) of *Pt*NHase and mutants were determined by the enzyme activity detection method mentioned above with a minor modification: the reaction time was changed to 2 min. The substrates were set to 5–7 gradient concentrations from 1 to 100 mM (1c), 2 to 200 mM (1f) and 0.05 to 5 mM (1h), respectively. Statistical analysis was performed by using the Graphpad Prism 7 software package (Graphpad Prism Software, San Diego, CA) *via* a Michaelis–Menten kinetic derivation.

### Analysis of thermal stability

The thermal stability of target enzymes containing wild-type *Pt*NHase and its mutants was determined using pre-incubated enzyme solutions similar to the reaction dosage described above without substrate at 50 °C for different durations. Residual enzyme activity was then measured under standard conditions, as described above. Relative activity was characterized by changes in the enzyme activity over time. The relative activity of 100% was represented by the catalytic activity of the enzyme solutions as the initial parameters.

### Purification and crystal structures

First, we obtained target cells without a Strep-tag through high-density fermentation using a 5 L fermentation tank.^18^ The cells were sonicated and resuspended in 10 mM KPB. After centrifugation, the supernatant was used to precipitate ammonium sulfate. The crude enzyme solution in the beaker was placed in an ice-water bath and constantly stirred. Ammonium sulfate was slowly added in the form of flow addition. The addition was stopped when ammonium sulfate saturation reached 40%, and the mixture was stirred continuously for 20–30 minutes. Crude enzyme supernatant was collected by centrifugation at 12 000*g* for 15–20 min at 4 °C and made up to the initial volume with KPB. As described above, the crude enzyme pellets were retained by centrifugation until ammonium sulfate saturation reached 60%. A crude enzyme solution was obtained by resuspension by adding a certain amount of KPB.

The crude enzyme samples of *Pt*NHase variants M46R and A129R were purified by the anion exchange method using a 5 mL DEAE column, and the enzymes were isolated with an AKTA purifier using a linear gradient of 0.3 to 0.5 M NaCl in buffer (10 mM KPB, 1 M NaCl). The enzyme solution was loaded onto a 6 mL Resource Q column (GE Healthcare UK Ltd) and separated with a linear gradient of 0.15 to 0.3 M NaCl in a buffer.

*Pt*NHase variants M46R and A129R were further purified by size exclusion chromatography using a Superdex 200 pg HiLoad 16/600 column (GE Healthcare UK Ltd) equilibrated with 10 mM KPB. The purified enzymes, M46R and A129R, were concentrated to 10 and 6 mg mL^−1^, respectively, using a 30 K Millipore Amicon Ultra tube. Sessile drop vapor diffusion crystallization experiments were set up in 192-well sessile drop culture plates (Shanghai Xinjiang Biotechnology Co., Ltd) by mixing 0.7 μL of the protein solution with 0.7 μL of the reservoir solution containing 1.0–1.4 M trisodium citrate, 0.1 M HEPES, pH 7–7.8, similar to the above previous crystallization of the wild-type *Pt*NHase. Both *Pt*NHase M46R and A129R crystals appeared after two days. Before data collection, crystals were soaked in a cryoprotectant solution containing 1.0 M trisodium citrate, 0.1 M HEPES, pH 7.5, and 50% glycerol. X-ray diffraction measurements for M46R and A129R were performed on a Bruker D8 VENTURE diffractometer.^39^ The diffraction data for M46R and A129R were indexed, integrated, and merged using the Proteum2 software suite. The crystals of M46R and A129R belonged to the same space group as that of wild-type *Pt*NHase, with nearly identical unit cell dimensions, allowing the wild-type crystal structure (PDB entry 1IRE) to be used directly for initial refinement and electron-density map calculations. Subsequently, the structures were adjusted using model-building to replace the side chains of the mutated residues. Model adjustment and refinement were conducted using Refmac5 and Coot.^40^ A summary of the data collection and model refinement statistics is provided in Table S5.† All protein structure figures were produced using PyMol (version 2.4, Schrödinger, https://pymol.sourceforge.net/).

### Molecular dynamics simulations

For the WT *Pt*NHase simulation, biological assembly 1 of 1IRE containing an αββα tetrameric crystal structure was used.^19^ βM46R and βA129R crystal structures with PDB codes 7W8L and 7W8M were used to simulate mutational variants. To obtain tetramers, structural alignment with 1IRE was performed. Before the simulation, protonation states were determined using the PROPKA tool installed in the PDB2PQR server.^41–43^ Next, the structures were solvated with at least 10 Å in each direction and 0.15 mol L^−1^ NaCl ions, and a neutralization option was added. The initial simulation box was 93 × 111 × 82 Å. For each variant, we performed a 200 ns Langevin molecular dynamics (MD) simulation at 300 K with a 1 fs time step under atmospheric pressure, with long-range electrostatic interactions calculated using particle mesh Ewald summation. Before the main simulation, 1 ns of water and ion equilibration, 1000 steps of energy minimization, and gradual heating up to 300 K for 60 ps were performed. The NAMD 2.14 code with the CHARMM27 force field was used.^44–47^ Parameters for the nonstandard active site with cobalt and posttranslationally oxidized cysteines were obtained based on extensive DFT/B3LYP/6-31G(d,p) and HF/6-31G* quantum calculations published in Peplowski's PhD thesis, used for the first time in SMD simulation for *Pt*NHase in 2008.^20^ Caver Analyst 2.0 BETA with a probe size of 2.0 Å was used in the analysis of cavities.^29^ DCCMs have been calculated in R with a bio3D plugin.^48^

## Data availability

All data included in this study are available upon request by contact with the corresponding author.

## Author contributions

Dong Ma, Zhongyi Cheng and Zhemin Zhou conceived the project and wrote the paper. Lukasz Peplowski wrote the part about MD simulations. Dong Ma, Zhongyi Cheng, Lukasz Peplowski, Laichuang Han, Yuanyuan Xia, Xiaodong Hou, Junling Guo, Dejing Yin, and Yijian Rao designed and performed all the experiments. Dong Ma, Zhongyi Cheng and Lukasz Peplowski analyzed the results.

## Conflicts of interest

The authors declare no competing interests.

## Supplementary Material

SC-013-D2SC02319A-s001
