# Supplementary material for: Insight into the broadened substrate scope of nitrile hydratase by static and dynamic structure analysis
Source: Chem Sci. 2022 Jul 6;13(28):8417–28. doi: 10.1039/d2sc02319a (PMC9297474; doi:10.1039/d2sc02319a)
Supplement: SC-013-D2SC02319A-s001 [file SC-013-D2SC02319A-s001.pdf]

Electronic Supplementary Material (ESI) for Chemical Science.
This journal is © The Royal Society of Chemistry 2022

Insight into the broadened substrate scope of nitrile hydratase by static and dynamic structure analysis

Dong Ma[1#], Zhongyi Cheng[1#], Lukasz Peplowski[2#], Laichuang Han[1], Yuanyuan Xia[1], Xiaodong Hou[1], Junling Guo[1], Dejing Yin[1], Yijian Rao[1*], Zhemin Zhou[1, 3*]

[1] Key Laboratory of Industrial Biotechnology (Ministry of Education), School of Biotechnology, Jiangnan University, Wuxi, Jiangsu, China

[2] Institute of Physics, Faculty of Physics, Astronomy and Informatics, Nicolaus Copernicus University in Torun, Grudziadzka 5, 87-100 Torun, Poland

[3] Jiangnan University (Rugao) Food Biotechnology Research Institute, Rugao, Jiangsu, China

[#] These authors contributed equally to this work.

*corresponding authors：

Yijian Rao,

School of Biotechnology, Jiangnan University

Wuxi, Jiangsu, 214122, China

E-mail: raoyijian@jiangnan.edu.cn

Zhemin Zhou,

School of Biotechnology, Jiangnan University

Wuxi, Jiangsu, 214122, China

E-mail: zhmzhou@jiangnan.edu.cn

Supporting Tables

Table S1: Applications of selected amides*.

Compound	Structure	Applications
Acrylamide	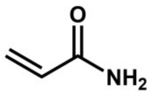	Being used in coagulators, soil conditioners and stock additives for paper treatment and paper sizing, and for adhesives, paints and petroleum recovering agents.[1]
Isobutyramide	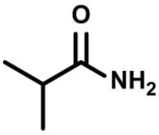	Being an important chemical intermediate employed in the organic dyes and pharmaceuticals, such as Ritonavir, an antiviral medicine that prevents HIV.[2]
Pentanamide	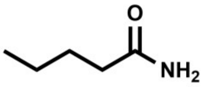	Building block for producing anxiolytic, antiepileptic and anesthetic agents.[3, 4]
Hexanamide	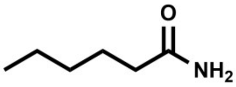	Building block in hexanamide derivatives used for HDACi reduced tumor growth.[5]
Pyrazinamide	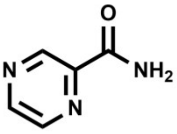	Being a mainstay in the multidrug regimens used to treat tuberculosis.[6]
Nicotinamide	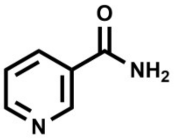	Precursor of NAD+ and to act in several tissues including skin, nervous system, and muscles. Playing a role in cancer prevention and therapy.[7]
Benzamide	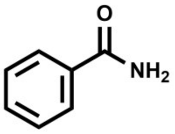	Being an intermediate used to synthesis medicines, dyes. Such as potential antimicrobial agents and inhibitor of acetylcholinesterase used for targeting Alzheimer's disease.[8]
Cinnamamide	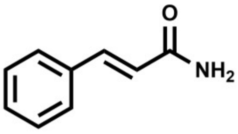	Privileged scaffold for designing and arriving at newly drug-like molecules with potential pharmacological activity, such as HDAC inhibitors and drug resistance reversal in neoplastic disease.[9]
1-Naphthamide	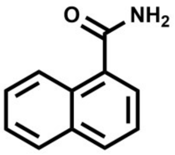	Precursor of anti-tumor agent.[10]
Sangivamycin	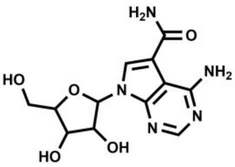	A purine nucleoside analogue possessing potent antitumor and antiretroviral activity.[11]

*Table includes refs 1-11.

Table S2: The primers used in this study.

Name	Primers (5’-3’)	Mutants
Pt-BF41A-F61	CATGTTTCCGGCAACCGCACGCGCAGGTTTTATG	F41A
Pt-BF41E-F61	CATGTTTCCGGCAACCGAACGCGCAGGTTTTATG	F41E
Pt-BF41H-F62	CATGTTTCCGGCAACCCATCGCGCAGGTTTTATG	F41H
Pt-BF41K-F61	CATGTTTCCGGCAACCAAACGCGCAGGTTTTATG	F41K
Pt-BF41L-F63	CATGTTTCCGGCAACCCTGCGCGCAGGTTTTATG	F41L
Pt-BF41Q-F61	CATGTTTCCGGCAACCCAGCGCGCAGGTTTTATG	F41Q
Pt-BF41-R62	GTTGCCGGAAACATGGCAAATGC	—
Pt-BM46A-F63	CAACCTTTCGCGCAGGTTTTGCAGGTCTGGATGAATTTCGTTTTG	M46A
Pt-BM46E-F65	CAACCTTTCGCGCAGGTTTTGAGGGTCTGGATGAATTTCGTTTTG	M46E
Pt-BM46F-F63	CAACCTTTCGCGCAGGTTTTTTCGGTCTGGATGAATTTCGTTTTG	M46F
Pt-BM46H-F63	CAACCTTTCGCGCAGGTTTTCATGGTCTGGATGAATTTCGTTTTG	M46H
Pt-BM46K-F66	CAACCTTTCGCGCAGGTTTTAAGGGTCTGGATGAATTTCG	M46K
Pt-BM46L-F66	CAACCTTTCGCGCAGGTTTTCTGGGTCTGGATGAATTTCG	M46L
Pt-BM46Q-F65	CAACCTTTCGCGCAGGTTTTCAGGGTCTGGATGAATTTCGTTTTG	M46Q
Pt-BM46C-F63	CAACCTTTCGCGCAGGTTTTTGTGGTCTGGATGAATTTCGTTTTG	M46C
Pt-BM46D-F63	CAACCTTTCGCGCAGGTTTTGATGGTCTGGATGAATTTCGTTTTG	M46D
Pt-BM46G-F63	CAACCTTTCGCGCAGGTTTTGGTGGTCTGGATGAATTTCGTTTTG	M46G
Pt-BM46I-F65	CAACCTTTCGCGCAGGTTTTATCGGTCTGGATGAATTTCGTTTTG	M46I
Pt-BM46N-F64	CAACCTTTCGCGCAGGTTTTAATGGTCTGGATGAATTTCGTTTTG	M46N
Pt-BM46P-F65	CAACCTTTCGCGCAGGTTTTCCGGGTCTGGATGAATTTCGTTTTG	M46P
Pt-BM46R-F63	CAACCTTTCGCGCAGGTTTTCGTGGTCTGGATGAATTTCGTTTTG	M46R
Pt-BM46S-F64	CAACCTTTCGCGCAGGTTTTAGTGGTCTGGATGAATTTCGTTTTG	M46S
Pt-BM46T-F64	CAACCTTTCGCGCAGGTTTTACCGGTCTGGATGAATTTCGTTTTG	M46T
Pt-BM46V-F64	CAACCTTTCGCGCAGGTTTTGTTGGTCTGGATGAATTTCGTTTTG	M46V
Pt-BM46W-F65	CAACCTTTCGCGCAGGTTTTTGGGGTCTGGATGAATTTCGTTTTG	M46W
Pt-BM46Y-F63	CAACCTTTCGCGCAGGTTTTTATGGTCTGGATGAATTTCGTTTTG	M46Y
Pt-BM46-R65	CTGCGCGAAAGGTTGCCGGAAAC	—

Pt-BL127A-F62	CGTTTATGGCGGCGCCCCGGCAAGCCGTG	L127A
Pt-BL127E-F63	CGTTTATGGCGGCGAACCGGCAAGCCGTG	L127E
Pt-BL127F-F63	GTTTATGGCGGCTTTCCGGCAAGCCGTG	L127F
Pt-BL127H-F63	GTTTATGGCGGCCATCCGGCAAGCCGTG	L127H
Pt-BL127K-F63	CGTTTATGGCGGCAAACCGGCAAGCCGTG	L127K
Pt-BL127Q-F68	GTTTATGGCGGCCAGCCGGCAAGCCGTG	L127Q
Pt-BP128A-F66	GTTTATGGCGGCCTGGCAGCAAGCCGTGAAGTTG	P128A
Pt-BP128E-F61	GTTTATGGCGGCCTGGAAGCAAGCCGTGAAGTTG	P128E
Pt-BP128F-F61	GTTTATGGCGGCCTGTTTGCAAGCCGTGAAGTTG	P128F
Pt-BP128H-F64	GTTTATGGCGGCCTGCATGCAAGCCGTGAAGTTG	P128H
Pt-BP128K-F61	GTTTATGGCGGCCTGAAAGCAAGCCGTGAAGTTG	P128K
Pt-BP128L-F68	GTTTATGGCGGCCTGCTGGCAAGCCGTG	P128L
Pt-BP128Q-F68	GTTTATGGCGGCCTGCAGGCAAGCCGTGAAG	P128Q
Pt-BA129E-F69	GTTTATGGCGGCCTGCCGGAAAGCCGTGAAGTTG	A129E
Pt-BA129F-F62	GTTTATGGCGGCCTGCCGTTTAGCCGTGAAGTTG	A129F
Pt-BA129H-F60	GTTTATGGCGGCCTGCCGCATAGCCGTGAAGTTG	A129H
Pt-BA129K-F64	GTTTATGGCGGCCTGCCGAAAAGCCGTGAAGTTG	A129K
Pt-BA129L-F63	GTTTATGGCGGCCTGCCGCTGAGCCGTGAAGTTG	A129L
Pt-BA129Q-F63	GTTTATGGCGGCCTGCCGCAGAGCCGTGAAGTTG	A129Q
Pt-BA129C-F62	GTTTATGGCGGCCTGCCGTGTAGCCGTGAAGTTG	A129C
Pt-BA129D-F65	GTTTATGGCGGCCTGCCGGATAGCCGTGAAGTTG	A129D
Pt-BA129G-F65	GTTTATGGCGGCCTGCCGGGTAGCCGTGAAGTTG	A129G
Pt-BA129I-F62	GTTTATGGCGGCCTGCCGATCAGCCGTGAAGTTG	A129I
Pt-BA129M-F62	GTTTATGGCGGCCTGCCGATGAGCCGTGAAGTTG	A129M
Pt-BA129N-F62	GTTTATGGCGGCCTGCCGAATAGCCGTGAAGTTG	A129N
Pt-BA129P-F66	GTTTATGGCGGCCTGCCGCCGAGCCGTGAAGTTG	A129P
Pt-BA129R-F62	GTTTATGGCGGCCTGCCGCGTAGCCGTGAAGTTG	A129R
Pt-BA129S-F62	GTTTATGGCGGCCTGCCGAGTAGCCGTGAAGTTG	A129S
Pt-BA129T-F65	GTTTATGGCGGCCTGCCGACCAGCCGTGAAGTTG	A129T

Pt-BA129V-F65	GTTTATGGCGGCCTGCCGGTTAGCCGTGAAGTTG	A129V
Pt-BA129W-F63	GTTTATGGCGGCCTGCCGTGGAGCCGTGAAGTTG	A129W
Pt-BA129Y-F62	GTTTATGGCGGCCTGCCGTATAGCCGTGAAGTTG	A129Y
Pt-BL127-A129-R64	GCCGCCATAAACGGCCTGATTCAC	—
Pt-WTNoStrep-F60	CAAAGCAGCAGCCGCATAAAAGGAGATATAGATATGACCG	WT
Pt-WTNoStrep-R60	CATATCTATATCTCCTTTTATGCGGCTGCTGCTTTGGTATC	—
PBAG-BS46A-F61	GCATCTGAAAGGTATTGCCTGGTGGGATAAAAGTCGCTTTTTC	S46A
PBAG-BS46E-F59	GCATCTGAAAGGTATTGAATGGTGGGATAAAAGTCGCTTTTTC	S46E
PBAG-BS46F-F61	GCATCTGAAAGGTATTTTCTGGTGGGATAAAAGTCGCTTTTTC	S46F
PBAG-BS46H-F61	GCATCTGAAAGGTATTCACTGGTGGGATAAAAGTCGCTTTTTC	S46H
PBAG-BS46K-F60	GCATCTGAAAGGTATTAAATGGTGGGATAAAAGTCGCTTTTTC	S46K
PBAG-BS46L-F61	GCATCTGAAAGGTATTCTCTGGTGGGATAAAAGTCGCTTTTTC	S46L
PBAG-BS46Q-F59	GCATCTGAAAGGTATTCAGTGGTGGGATAAAAGTCGCTTTTTC	S46Q
PBAG-BS46Y-F59	GCATCTGAAAGGTATTTATTGGTGGGATAAAAGTCGCTTTTTC	S46Y
PBAG-BS46-R60	CTTTCAGATGCATCCAGGTCAGAATAC	—
PBAG-BL133A-F63	GCATAGCGCAGCCCTGCCGGGTGCCGAAC	L133A
PBAG-BL133E-F60	CGCATAGCGAAGCCCTGCCGGGTGC	L133E
PBAG-BL133F-F62	CGCATAGCTTTGCCCTGCCGGGTGC	L133F
PBAG-BL133H-F64	CGCATAGCCATGCCCTGCCGGGTGC	L133H
PBAG-BL133K-F60	CGCATAGCAAAGCCCTGCCGGGTGC	L133K
PBAG-BL133Q-F64	GCATAGCCAGGCCCTGCCGGGTG	L133Q
PBAG-BL133Y-F60	CGCATAGCTATGCCCTGCCGGGTGC	L133Y
PBAG-BL133-R62	CTATGCGGTTCATGCAGGCGTTC	—
PCalt-BY46A-F57	CAAGGTCTGGCCAATTTAGATGAGTTCCGCC	Y46A
PCalt-BY46E-F55	CAAGGTCTGGAGAATTTAGATGAGTTCCG	Y46E
PCalt-BY46F-F59	GTCAAGGTCTGTTCAATTTAGATGAGTTCCGC	Y46F
PCalt-BY46H-F54	CAAGGTCTGCACAATTTAGATGAGTTCC	Y46H
PCalt-BY46K-F57	CAAGGTCTGAAAAATTTAGATGAGTTCCGCC	Y46K
PCalt-BY46L-F60	GTCAAGGTCTGTTAAATTTAGATGAGTTCCGCCATGC	Y46L
PCalt-BY46Q-F60	GTCAAGGTCTGCAAAATTTAGATGAGTTCCGCCATGC	Y46Q

PCalt-BY46-R61	CAGACCTTGACCGGCGGTAC	—
PCalt-BT128A-F62	GTGGGTGTTAGCGCAGTGCGCGAAGTTAGTAG	T128A
PCalt-BT128E-F61	GTGGGTGTTAGCGAAGTGCGCGAAGTTAGTAGCG	T128E
PCalt-BT128F-F61	GTGGGTGTTAGCTTCGTGCGCGAAGTTAGTAG	T128F
PCalt-BT128H-F61	GTGGGTGTTAGCCACGTGCGCGAAGTTAGTAG	T128H
PCalt-BT128K-F60	GTGGGTGTTAGCAAAGTGCGCGAAGTTAGTAG	T128K
PCalt-BT128L-F61	GTGGGTGTTAGCCTGGTGCGCGAAGTTAGTAGCG	T128L
PCalt-BT128Q-F61	GTGGGTGTTAGCCAGGTGCGCGAAGTTAGTAGCG	T128Q
PCalt-BT128Y-F61	GTGGGTGTTAGCTATGTGCGCGAAGTTAGTAGCG	T128Y
PCalt-BT128-R59	GCTAACACCCACCTTAATAACTTGCTC	—

Table S3: Liquid phase detection conditions and reaction conditions catalyzed by *Pt*NHase for different products.

Products	RT[b]（min）	λ（nm）	Substrates	RT[b]（min）	Concentration（mM）	RT[a]（min）	Flow Rate (mL·min^{-1})
Acrylamide	4	215	1a	7.5	100	5	0.6
Isobutyramide	4.5-4.8	202	1b*	-	200	10	0.6
Pentanamide	6	202	1c*	-	100	10	0.6
Hexanamide	9.5	202	1d*	-	20	5	0.6
Pyrazinamide	4	261	1e	7.5	100	10	0.6
Nicotinamide	4	215	1f	7.5	200	10	0.6
1-Naphthamide	12	215	1i**	-	1	5	0.6
Sangivamycin	3.55	278	1k	4	1	600	0.6
Benzamide	3.5-4	215	1g*	17	50	5	1
Cinnamamide	6.0-7.0	261	1h*	37	5	5	1
Thiacloprid-amide	4.5-5	242	1j*	9.0-10.0	0.79	5	1

RT[a] represents reaction time in enzyme activity assay. RT[b] refers to retention time in liquid phase detection. *represents that the substrate solution was prepared with 10 mM KPB containing 10% methanol. **indicates that the substrate solution was prepared with 10 mM KPB containing 15% methanol.

Table S4: Kinetic parameters of wild-type *Pt*NHase and its mutants toward substrate 1c, 1f and 1h.

Substrate	Mutant	K_m (mM)	k_{cat} (s^{-1})	k_{cat}/K_m ($s^{-1}mM^{-1}$)
	WT	9.4±0.7	123.7±36	13.1
1c	M46R	9.3±1.8	351.9±100	37.8
	A129R	7.8±0.7	138.7±30	17.8
	WT	5.2±0.50	544.5±12	104.7
1f	M46R	2.4±0.26	1715.6±32	714.9
	A129R	3.1±0.60	2061.6±76	665.0
	WT	0.50±0.16	119.9±11	237.8
1h	M46R	0.49±0.05	419.9±12	849.8
	A129R	0.50±0.06	400.8±14	805.9

1c, 1f and 1h refers to pentanenitrile, 3-cyanopyridine and cinnamonitrile, respectively.

Table S5: Data collection and refinement statistics for obtained crystal structures.

**Data Collection**	**M46R**	**A129R**
Space group	P 32 2 1	P 32 2 1
Cell dimensions: a, b, c (Å)	65.46, 65.46, 182.81, 90, 90, 120	65.52, 65.52, 183.75, 90.00, 90.00, 120.00
Resolution (Å)	24.09 - 2.3 (2.382 - 2.3)	21.45 - 2.6 (2.693 - 2.6)
Rsym or Rmerge	0.087 (0.389)	0.090 (0.297)
I / σI	15.3 (2.8)	14.1 (4.3)
Completeness (%)	96.02 (90.50)	98.60 (99.44)
Redundancy	5.9 (3.0)	4.4 (3.4)
	**Refinement**	
No. reflections all/free	20173 / 1868	14547 / 1418
R-factor / R-free	0.1834 (0.2462) / 0.2348 (0.3225)	0.1820 (0.2122) / 0.2405 (0.3009)
No. protein atoms	3651	3580
No. of water molecules	197	103
No. of cobalt ions	1	1
r.m.s. deviations		
Bond lengths (Å)	0.009	0.008
Bond angles (°)	0.94	1.02
Ramachandran outliers (%)	0	0
PDB accession code	7W8L	7W8M

[a] Values in parentheses are for highest-resolution shell.

Supporting Figures

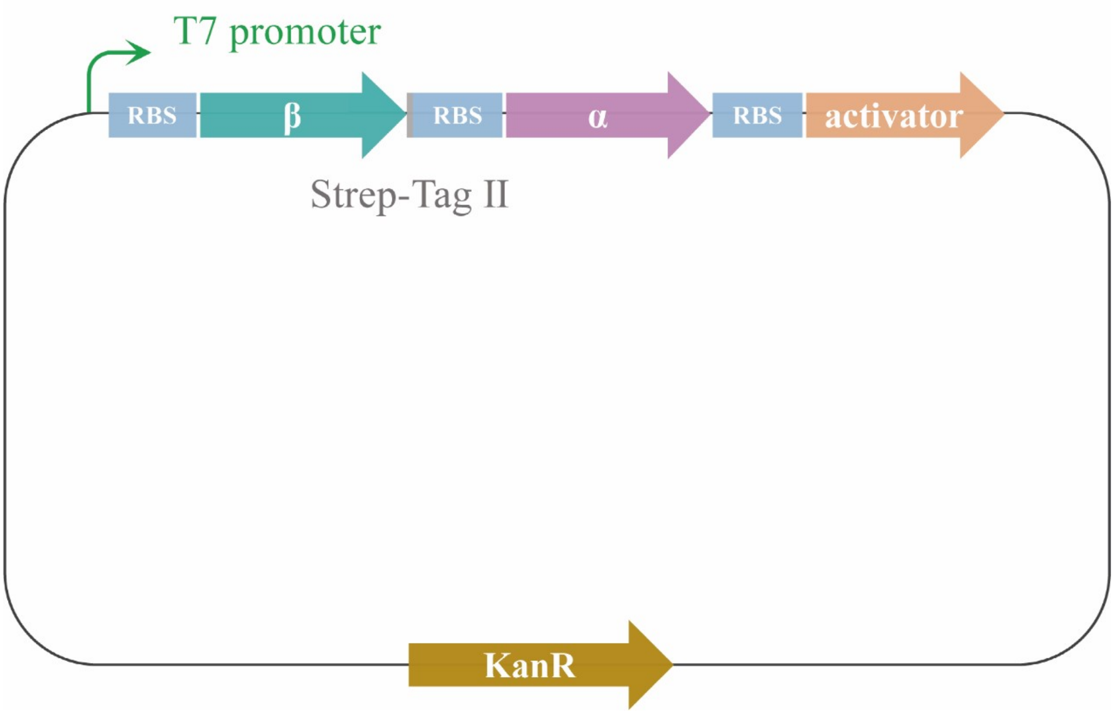

Figure S1: An expression plasmid used for expressing *Pt*NHase containing alpha, beta subunit with pET-24a as the vector. RBS refers to ribosome binding site. KanR means aminoglycoside phosphotransferase containing resistance to kanamycin.

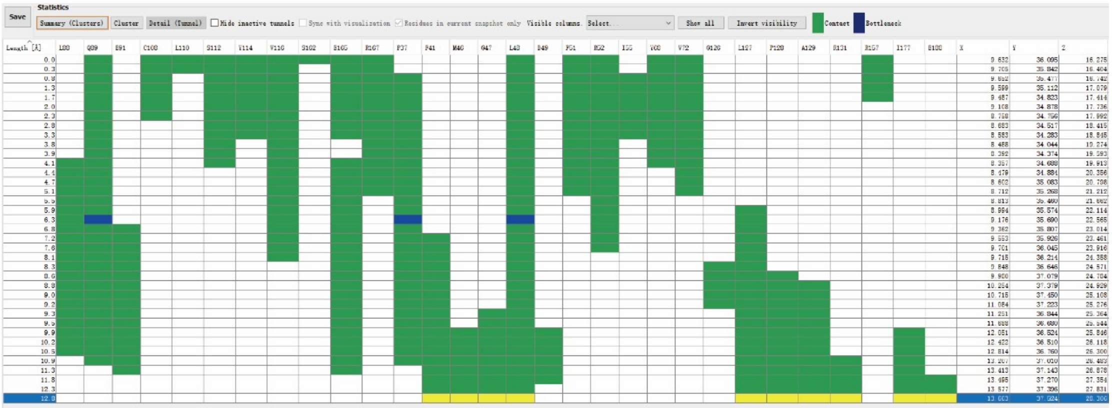

Figure S2: Substrate access tunnel analysis of WT *Pt*NHase. The distance from amino acid forming the substrate access tunnel to the active site of *Pt*NHase is calculated in CAVER Analyst 2.0. The entrance of substrate access tunnel are formed by ten amino acid residues marked in yellow that locate at the protein surface. The amino acid residues marked in blue are the bottleneck residues of the tunnel.

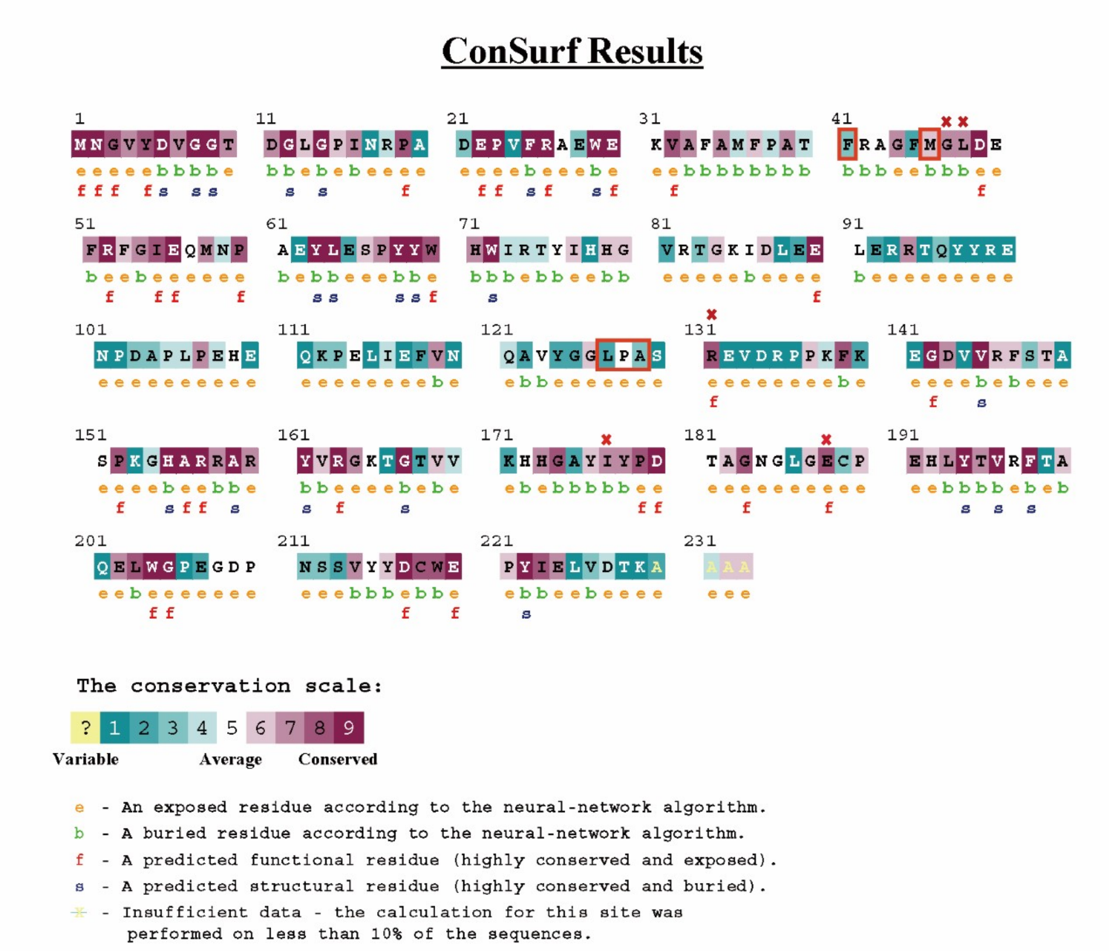

Figure S3: Conservative prediction of amino acid residues at the β subunit of *Pt*NHase. The consurf database provides pre-calculated evolutionary conservation profiles for proteins of known structure in the PDB.

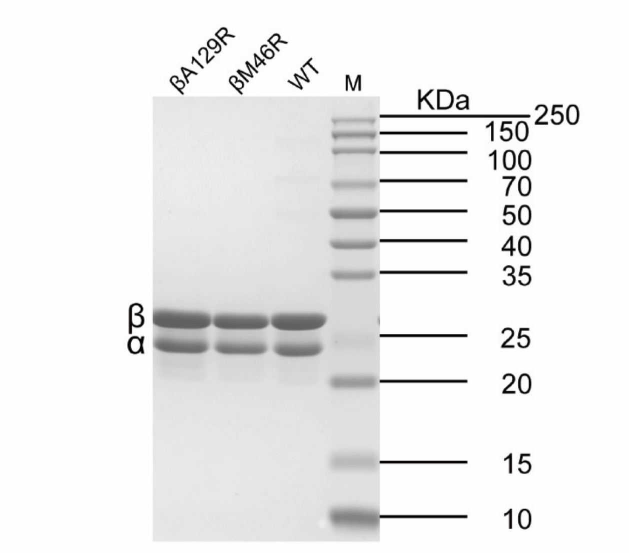

Figure S4: SDS-PAGE of purified *Pt*NHase and its mutants.

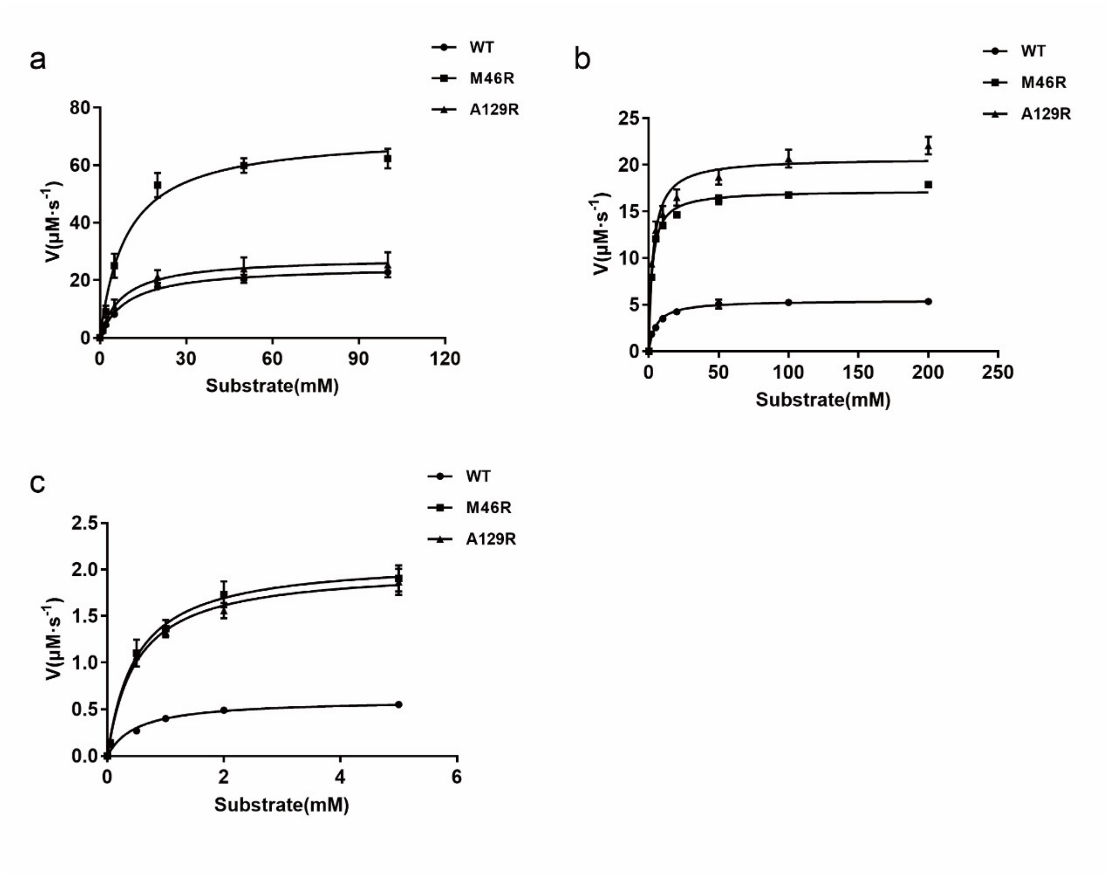

Figure S5: The kinetic plots of *Pt*NHase and its mutants.

(a) 1c: Pentanenitrile; (**b)** 1f: 3-Cyanopyridine; (**c)** 1h: Cinnamonitrile.

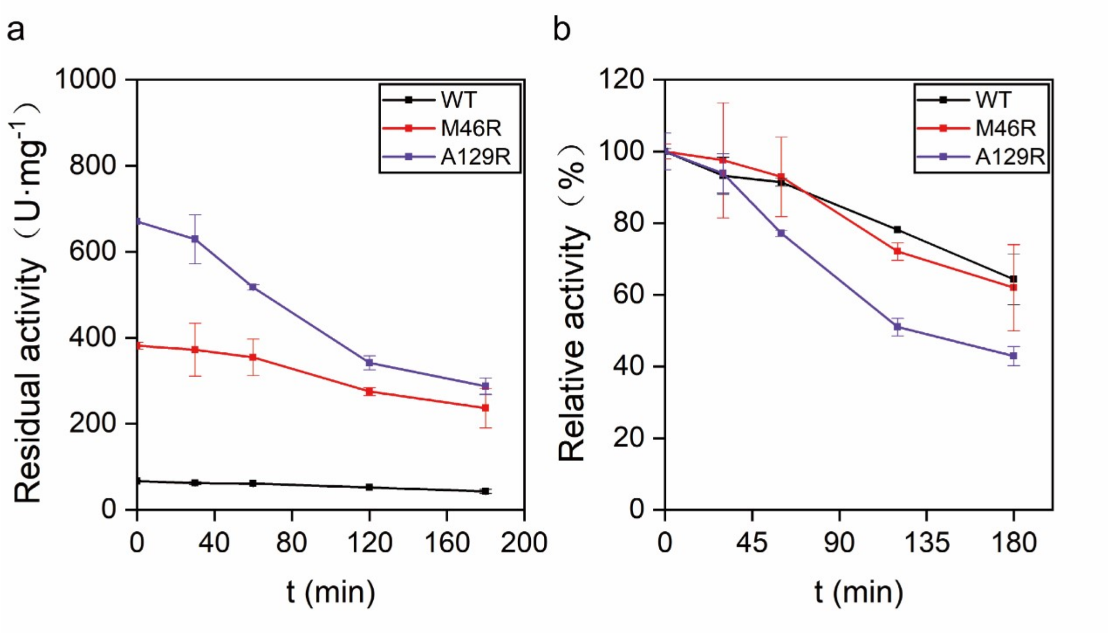

Figure S6: Determination of the half-life time of the WT *Pt*NHase and its mutants.

(a) Residual activity was detected over time at 50 °C.

(b) Thermal stability is characterized by statistic and analysis of relative activity.

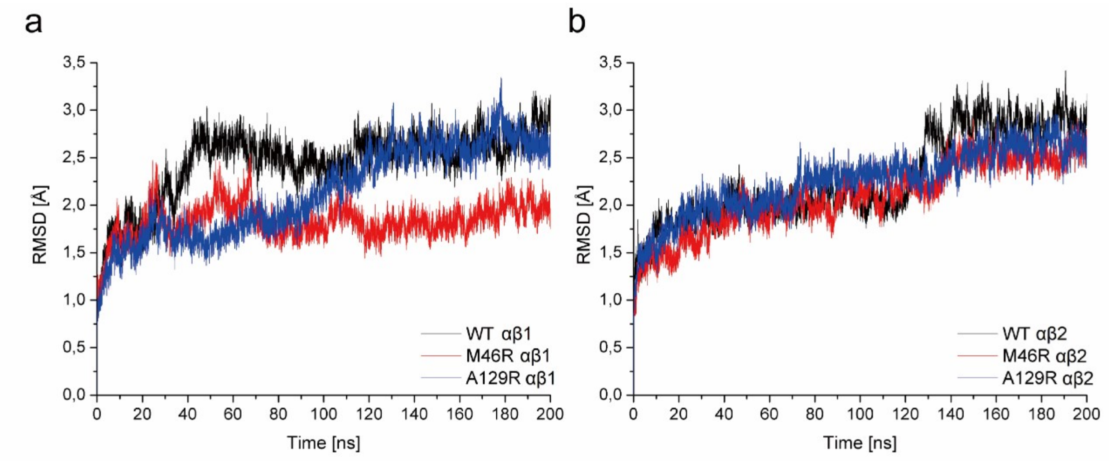

Figure S7: Root Mean Square Deviations (RMSD) plots of both *Pt*NHase dimers.

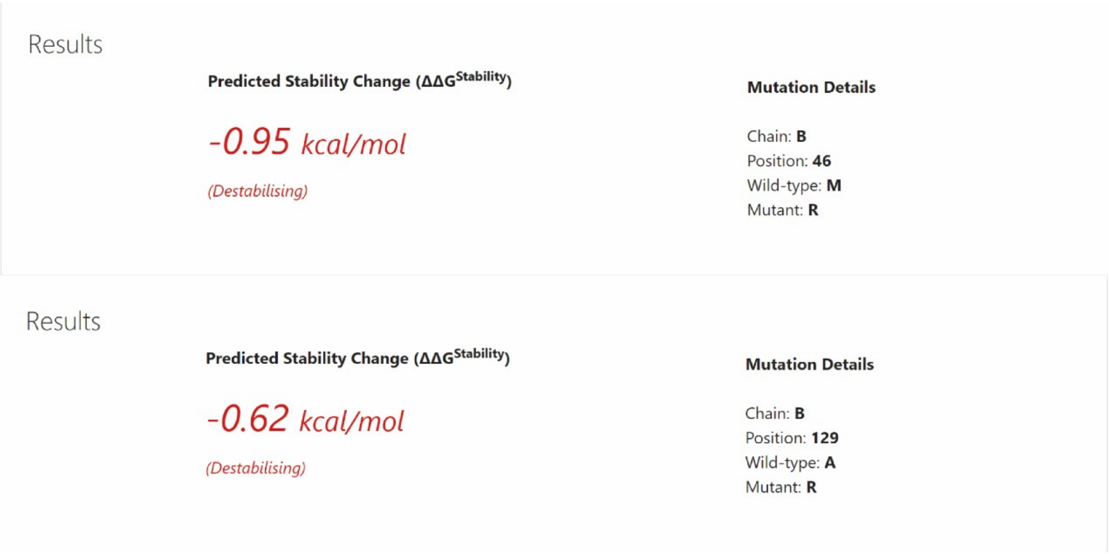

Figure S8: Gibbs Free Energy (ΔΔG) calculation for *Pt*NHase-βM46R and *Pt*NHase-βA129R based on DynaMut2.

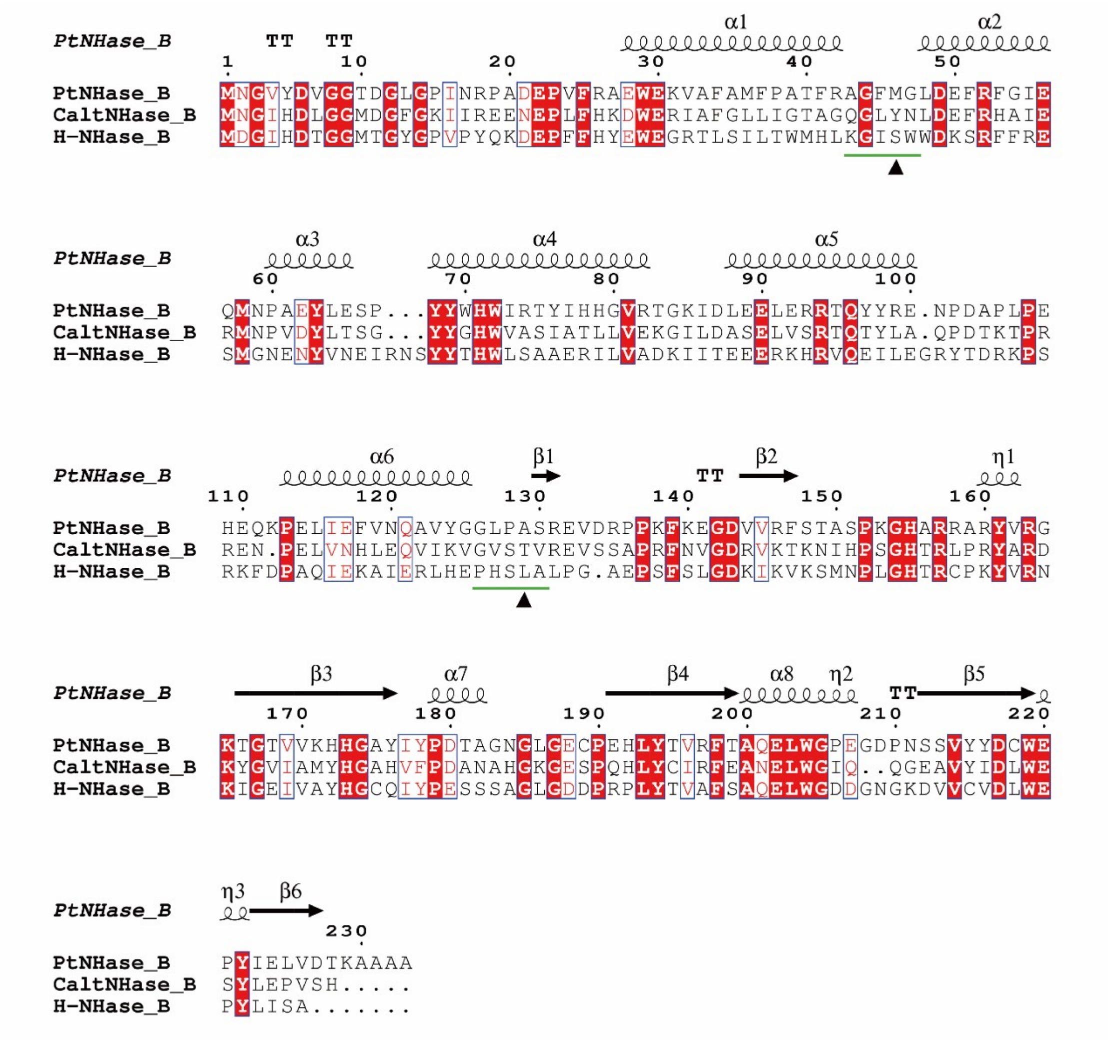

Figure S9: Amino acid sequence alignment between the β subunit of *Pt*NHase, *Calt*NHase and H-NHase. The alignment is conducted by using ClustalW. The graph is prepared using Espript server (Espript3.0, https://espript.ibcp.fr/ESPript/ESPript/).

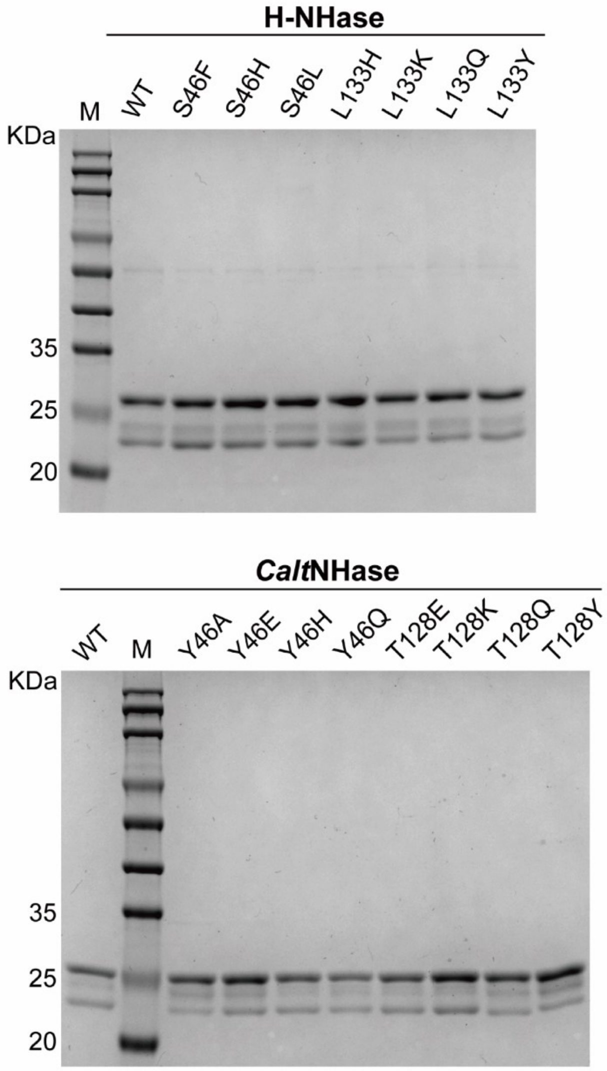

Figure S10: SDS-PAGE analysis of H-NHase and *Calt*NHase.

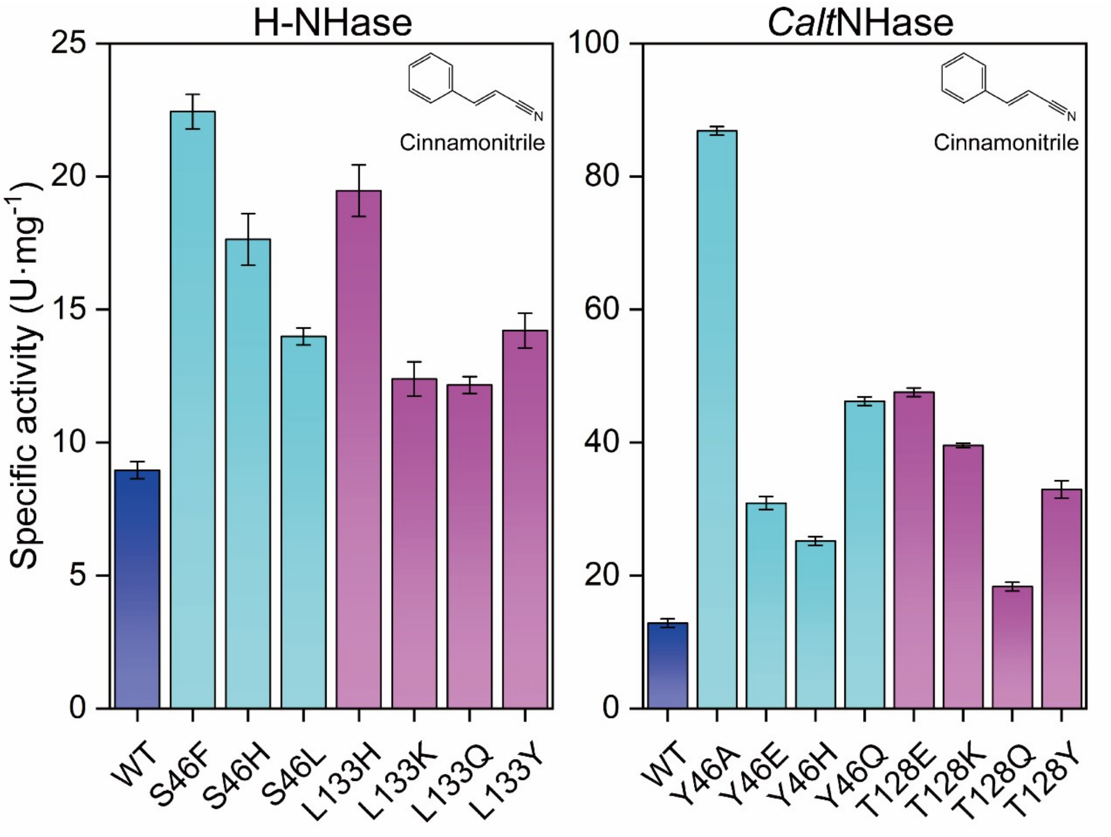

Figure S11: Catalytic activity of H-NHase and *Calt*NHase together with their positive mutants toward cinnamonitrile.

Amino acid sequences

Beta subunit

MNGVYDVGGTDGLGPINRPADEPVFRAEWEKVAFAMFPATFRAGFMGLDEFRFGIEQM
NPAEYLESPYYWHWIRTYIHHGVRTGKIDLEELERRTQYYRENPDAPLPEHEQKPELIEFV
NQAVYGGLPASREVDRPPKFKEGDVVRFSTASPKGHARRARYVRGKTGTVVKHHGAYI
YPDTAGNGLGECPEHLYTVRFTAQELWGPEGDPNSSVYYDCWEPYIELVDTKAAAA

Alpha subunit

MTENILRKSDEEIQKEITARVKALESMLIEQGILTTSMIDRMAEIYENEVGPHLGAKVVVK
AWTDPEFKKRLLADGTEACKELGIGGLQGEDMMWVENTDEVHHVVVCTLCSCYPWPV
LGLPPNWFKEPQYRSRVVREPRQLLKEEFGFEVPPSKEIKVWDSSSEMRFVVLPQRPAGT
DGWSEEELATLVTRESMIGVEPAKAVA

The two amino acid residues marked in red are the mutation sites βM46 and βA129.

Reference

1. H. Yamada and M. Kobayashi, *Biosci. Biotechnol. Biochem.*, 1996, **60**, 1391-1400.
2. K. Wu and Y. Li, *Journal of Molecular Liquids*, 2020, **311**, 113294.
3. A. Haj-Yehia and M. Bialer, *Journal of pharmaceutical sciences*, 1988, **77**, 831-834.
4. H. Oyasu, M. Nagano, A. Akahane, M. Tomoi, T. Tada and M. Matsuo, *Journal of medicinal chemistry*, 1994, **37**, 1378-1381.
5. T. V. S. Shankar, B. Sulka, P. Hubert, R. Hubaux, P. Delvenne, H. Pendeville-Samain, I. Boittiaux, D. M. Lambert, J. Wouters, C. Maillard, S. Blacher, A. Noël, I. Struman and L. Willems, *Journal of Cancer Sciences*, 2015, **2**.
6. Q. Sun, X. Li, L. M. Perez, W. Shi, Y. Zhang and J. C. Sacchettini, *Nat. Commun.*, 2020, **11**, 339.
7. F. Scatozza, F. Moschella, D. D'Arcangelo, S. Rossi, C. Tabolacci, C. Giampietri, E. Proietti, F. Facchiano and A. Facchiano, *J. Exp. Clin. Cancer Res.*, 2020, **39**, 211.
8. M. N. Joy, V. A. Bakulev, Y. D. Bodke and S. Telkar, *Pharmaceutical Chemistry Journal*, 2020, **54**, 604-621.
9. N. Gaikwad, S. Nanduri and Y. V. Madhavi, *Eur. J. Med. Chem.*, 2019, **181**, 111561.
10. Y. Lv, M. Li, T. Liu, L. Tong, T. Peng, L. Wei, J. Ding, H. Xie and W. Duan, *ACS Med. Chem. Lett.*, 2014, **5**, 592-597.
11. N. G. Dolloff, J. E. Allen, D. T. Dicker, N. Aqui, D. Vogl, J. Malysz, G. Talamo and W. S. El-Deiry, *Mol. Cancer Ther.*, 2012, **11**, 2321-2330.
